# Factor Analysis of Genetic Parameters for Body Conformation Traits in Dual-Purpose Simmental Cattle

**DOI:** 10.3390/ani12182433

**Published:** 2022-09-15

**Authors:** Lei Xu, Hanpeng Luo, Xiaoxue Zhang, Haibo Lu, Menghua Zhang, Jianjun Ge, Tao Zhang, Mengjie Yan, Xueting Tan, Xixia Huang, Yachun Wang

**Affiliations:** 1 College of Animal Science, Xinjiang Agricultural University, Urumqi 830052, China; 2 College of Animal Science and Technology, China Agricultural University, Beijing 100193, China; 3 Beijing SUNLON Biological Seed Industry Innovation Technology Limited Company, Beijing 101206, China; 4 Xinjiang Hutubi Farm, Changji 831200, China

**Keywords:** Simmental cattle, genetic parameter, body conformation traits, factor analysis

## Abstract

**Simple Summary:**

Body conformation traits are closely related to economically important characteristics and should be considered in cattle breeding programs. A variety of body conformation traits recorded by classifiers can complicate the analysis process. Factor analysis can reduce the number of variables by combining two or more variables into a single factor, which has biological significance. The results of this study could be used by breeders to define conformation indexes and implement genetic assessments for conformation traits in dual-purpose breeds.

**Abstract:**

In this study, we estimated the genetic parameters for 6 composite traits and 27 body conformation traits of 1016 dual-purpose Simmental cattle reared in northwestern China from 2010 to 2019 using a linear animal mixed model. To integrate these traits, a variety of methods were used as follows: (1) genetic parameters estimates for composite and individual body conformation traits based on the pedigree relationship matrix (A) and combined genomic-pedigree relationship matrix (H); (2) factor analysis to explore the relationships among body conformation traits; and (3) genetic parameters of factor scores estimated using A and H, and the correlations of EBVs of the factor scores and EBVs of the composite traits. Heritability estimates of the composite traits using A and H were low to medium (0.07–0.47). The 24 common latent factors explained 96.13% of the total variance. Among factors with eigenvalues ≥ 1, F1 was mainly related to body frame, muscularity, and rump; F2 was related to feet and legs; F3, F4, F5, and F6 were related to teat placement, teat size, udder size, and udder conformation; and F7 was related to body frame. Single-trait analysis of factor scores yielded heritability estimates that were low to moderate (0.008–0.43 based on A and 0.04–0.43 based on H). Spearman and Pearson correlations, derived from the best linear unbiased prediction analysis of composite traits and factor scores, showed a similar pattern. Thus, incorporating factor analysis into the morphological evaluation to simplify the assessment of body conformation traits may improve the genetics of dual-purpose Simmental cattle.

## 1. Introduction

Dual-purpose Simmental cattle, a popular breed, exhibit high milk and meat production, good fertility and profitability [1,2,3,4]. Dual-purpose Simmental cattle were introduced to Northwest China in the 1950s. Core breeding farms have resulted in improvements in more than 400,000 cattle in China over several years [5]. These core breeding farms record milk production, milk composition, body measurement, and body weight every month. The breeding goals for dual-purpose Simmental cattle in Northwest China are milk production, milk quality, and growth traits. The average 305-day milk yield reached 5469 kg, the average fat content was 4.13%, the protein content was 3.33% [3], and the average daily weight gain from birth to 24 months of age was 0.70 kg·d^−1^ [6].

Body conformation traits are closely related to economically important traits, such as milk production [7], reproduction [8], health [9], profitability [10], and lifespan [11]. Therefore, studying the genetics of body conformation traits is as important as other production traits from an economic perspective. Understanding the genetic parameters of body conformation traits is crucial for implementing breeding programmes. Multiple regression has been used to analyze the relationship between body conformation traits, and the results have shown that the traits are correlated genetically and phenotypically [12]. For instance, Simčič et al. [12] found high genetic and phenotypic correlations between body frame traits in first parity Rendena cows. In some other cattle breeds, VanRaden et al. [13] and Mazza et al. [14], found strong genetic correlations between rear udder height and rear udder width, with values ranging from 0.85 to 0.95. Using a large number of traits with common information in multiple regression can lead to biased estimates of their relationship with productive traits [15]. Factor analysis is a useful multivariate technique for analyzing correlated traits, and it can remove redundant information introduced by incorporating multiple variables [16,17]. Mazza et al. [18] and Olasege et al. [19] suggested the use of latent factors in genetic evaluation, avoiding the analysis of highly correlated traits, thereby improving precision and reducing computational burdens for large datasets.

To date, a linear identification of body conformation traits of dual-purpose Simmental cattle in Northwest China has not been conducted, and breeding programmes do not include linear-type traits. This study aims to estimate genetic parameters for body conformation traits in dual-purpose Simmental cattle using factor analysis. The results could be used to develop a national genetic evaluation framework for the improvement of body conformation traits for dual-purpose Simmental cattle in China.

## 2. Materials and Methods

Approval from the local Institutional Animal Care and Use Committee was not required for this study because the data were obtained by field measurements, and no animal experiments were conducted.

### 2.1. Phenotypic and Pedigree Data

In 2020, our team applied for a dual-purpose cattle type classification project. After deliberation, we identified 6 composite traits and 27 body conformation traits. There were 17 measured traits and 10 scored traits. The measured traits included stature (ST), body depth (BD), chest width (CW), withers width (WW), hind leg half circumference (HLHC), rear leg height (RLH), rump length (RL), rump width (RW), rump angle (RA), rear udder height (RUH), rear udder width (RUW), median suspensory (MS), udder depth (UD), fore udder length (FUL), front teat length (FTL), front teat diameter (FTD), and heel depth (HD). The scored traits included ribs and bone (RB), rear legs side view (RLSV), bone quality (BQ), foot angle (FA), rear legs rear view (RLRV), fore udder attachment (FUA), rear udder length (RUL), udder balance (UB), fore teat placement (FTP), and rear teat placement (RTP). The individual body conformation traits are specific to certain body regions of the animals, including the body frame, muscularity, rump, feet and legs, and mammary system. The six composite traits summarized body frame, muscularity, rump, feet and legs, mammary system, and final score.

Conformation traits of 1200 dual-purpose Simmental cattle born from 2010 to 2019 were measured at Xinjiang Hutubi Farm, Kekedala Chuangjin Farm, and Xinjiang Haozi Animal Husbandry Farm in Northwest China. After quality control was applied using the threshold of the mean ± three times the standard deviation, 1016 conformation records remained and were used for analysis. The pedigree file used for the analysis included data for 1988 animals, with each animal being traced back three generations. In the full datasets, one sire had a maximum of 195 offspring with records, whereas 16 sires had only one offspring. More than 62 dams had 2 or more offspring.

### 2.2. Genotype Data

The Illumina 100K Bovine BeadChip was used to genotype 516 Simmental cows. For analysis, common SNPs were obtained from 100K bead chips as target files. Quality control of the SNP genotyping was carried out with PLINK 1.07 software (Boston, MA, USA) [20]. All genotyped animals had a call rate greater than 0.90. SNPs were removed if the call rate was less than 0.90 and the minor allele frequency (MAF) was less than 0.01. After quality control, data for 88,913 SNPs from 516 animals remained for analysis.

### 2.3. Genetic Connectedness

To estimate genetic parameters, the genetic connectedness of the animals in the dataset must be determined. If the breeding process affects the genetic connectedness of cattle at different farms over time, this change would be reflected in a change in the average relatedness in birth-year cohorts and among the populations at different farms. The indirect method of Sargolzaei [21], implemented in the software package CFC, was used to compute the coefficient of relationship among animals.

### 2.4. Variance Component Estimates for Body Conformation Traits

The single-trait animal model was used to estimate genetic and residual variance for the 6 composite traits and 27 individual body conformation traits with the average information-restricted maximum likelihood (AI-REML) method. The AIREMLF90 procedure of BLUPF90 1.0.1 software (Athens, GA, USA) was used [22]. The animal linear mixed model for the single trait analysis was as follows:

(1) Y=Xβ+Za+e

where ***Y*** indicates the vector of 6 composite traits and 27 individual body conformation traits; β is the vector of the fixed effects, including herd-year of evaluation (5 different levels), days in milk (ten classes: from 10 to 30 days after calving, from 31 to 270 days after calving in 30-day intervals, and >270 days after calving), age at first calving (seven classes: <23 months, from 23 to 34 months in 2-month intervals, and >34 months), and parity (four classes: 1, 2, 3, and ≥4); a is the vector of random animal additive genetic effects; e is the vector of random residual effects; and X, and Z are the incidence matrices assigning observations to fixed and random animal effects.

The genetic effect was modeled using two kinds of genetic variance-covariance matrices: the pedigree relationship matrix (A) [23], and the combined genomic-pedigree relationship matrix (H) [24].

The heritability and standard error were estimated according to Austin Putz et al. [25] using the following formula:

(2) $$h^{2}=\frac{\sigma_{a}^{2}}{\sigma_{a}^{2}+\sigma_{e}^{2}}$$

(3) $$SE\left(h^{2}\right)=\left(\frac{h^{2}\left(1-h^{2}\right)}{a}-\frac{h^{2}\left(h^{2}\right)}{a}\right)\left(\begin{array}{cc} var\left(a\right) & cov\left(a,e\right)\\ cov\left(e,a\right) & var\left(e\right) \end{array}\right)\left(\begin{array}{c} \frac{h^{2}\left(1-h^{2}\right)}{a}\\ -\frac{h^{2}\left(h^{2}\right)}{a} \end{array}\right)$$

### 2.5. Factor Analysis

Factor analysis was performed using the FACTOR procedure in SAS 8.0 software (Cary, NC, USA). In this analysis, a set of *n* observation variables ($y_{1},\ldots .,y_{n}$) is synthesized into a new set of *p* (*p* < *n*) latent variables ($X_{1},\ldots .,X_{p}$), which are referred to as common latent factors. As described by Kaiser (1960), varimax rotation was used to maintain the orthogonality of the extracted factors. Only components with eigenvalues≥1 were retained for the analyses (i.e., the Kaiser criterion; Russel [26], Mazza et al. [18], Olasege et al. [19]). By observing the individual body conformation trait loadings, the analysis was interpreted from a biological point of view. Based on the standardized scoring coefficients, we calculated the sample scores for each animal. According to Russel [27], the classic factor analysis equation specifies that a measure being factored can be represented by the following equation accounting for n factors:

(4) $$X_{m}=W_{m1}F_{1}+W_{m2}F_{2}+\ldots W_{mn}F_{n}+W_{mn}U_{n}+e_{m}$$

where $X_{m}$ is the m-th measure, $F_{n}$ is the *n*-th common factor that underlies the m-th measure being analyzed, and $U_{n}$ is the *n*-th factor that is unique to each m-th measure. Furthermore, $W_{mn}$ represents the n-th factor loading coefficients or loadings of each m-th measure on the respective factors, whereas $e_{m}$ reflects the random measurement errors in each m-th measure. Using this equation, we can divide the variance in the measure being factored into three parts. The first part of the variance of the measure reflects the impact of the common factors, the second part reflects the influence of the unique factor associated with the measure, and the third is the variance of the random error [26].

### 2.6. Estimation of Genetic Parameters Using Factor Analysis

In the single-trait animal model presented above, genetic parameters were estimated by fitting the factor scores as y. The estimated breeding values (EBVs) of the factor scores were then subjected to rank correlation analysis with the EBVs of the composite traits using SPSS.

## 3. Results

### 3.1. Phenotype

The descriptive statistics for the 6 composite traits and 27 individual body conformation traits, including the mean, standard deviation, minimum, maximum, and coefficient of variation are summarized in Table 1. In the composite traits, the coefficient of variation ranged from 2.52% (final score) to 7.73% (rump). For the individual body conformation traits, the coefficient of variation ranged from 3.20% (stature) to 77.16% (udder depth).

### 3.2. Genetic Connectedness

**Figure 1:**
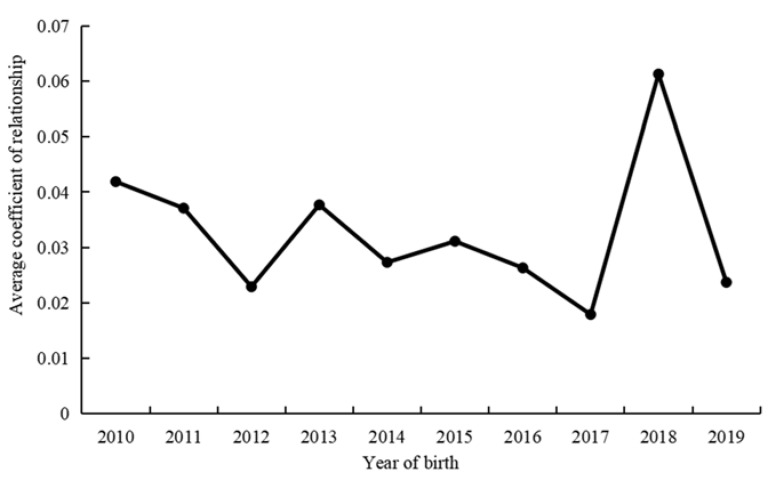
Evolution of average coefficient of relationship between three farms subpopulations according to the year of birth of Simmental cattle.

All three farms use artificial insemination for breeding. Figure 1 shows the trend of the average coefficient of relationship by year of birth of Simmental cattle. The relationship coefficient among Simmental cattle born on the three farms from 2010 to 2019 ranged from 0.0179 to 0.0613, with irregular variation. Of the annual changes in the coefficient of relationship, the largest change was from 2017 to 2018.

### 3.3. Heritability of Conformation Traits

The heritability estimates for the 6 composite traits and 27 individual body conformation traits are presented in Table 2. Using the pedigree relationship matrix (A), the estimates for the composite traits ranged from 0.07 (muscularity composite) to 0.43 (body frame composite); using the combined genomic-pedigree matrix (H), the estimates for the composite traits ranged from 0.10 (muscularity composite) to 0.47 (body frame composite). The estimation for the final score was 0.18 from A and 0.14 from H. In general, the highest estimates of heritability were obtained in body frame traits, whereas the estimates for muscularity traits and feet and legs traits were low. For the individual body conformation, the heritability estimates from A ranged from 0.05 (HLHC and RAB) to 0.56 (ST), and from H, they ranged from 0.03 (rear udder length; RUL) to 0.65 (ST). The standard errors of heritability estimates were all ≤ 0.10, except for those for ST and RL. In addition, estimates of other body conformation traits from A and H were very similar. There was little improvement in the accuracy of the estimated heritability, i.e., the standard errors of the two models were similar. However, the average heritability estimated by H was higher than that of A, except for individual muscularity traits.

### 3.4. Factor Analysis

The eigenvalues and the proportion of total and cumulative variance explained by each factor are listed in Table 3. The 24 factors after varimax rotation explained 96.13% of the total variation among the 27 individual body conformation traits. The first factor (F1) accounted for the largest proportion (13.51%) of the total variability. The first 9 factors with eigenvalues ≥ 1 were retained for further analysis. The varimax rotated factor patterns coefficients and commonalities are reported in Table 4. Only loading coefficients ≥$\;\left|0.40\right|$ [27] were reported for each body conformation trait. F1 was heavily loaded for ST (0.65), BD (0.46), HLHC (0.60), RL (0.72), and RW (0.57). F1 accounted for traits belonging to the body frame, muscularity, and rump. Factor 2 (F2), with 8.13% variability, had higher loadings on RLH (0.46), HD (0.76), and FA (0.77), whereas the third factor (F3) accounted for 7.32% of the proportional variability and loaded heavily on FTP (0.79) and RTP (0.73). The fourth factor (F4) explained 6.01% of the variability and loaded heavily on FTL (0.80) and FTD (0.81), whereas the fifth factor (F5) explained 5.18% of the variability and had higher loadings on FUL (0.70). F6 accounted for 4.93% of the proportional variability and higher loadings for UB (0.65), whereas F7 accounted for 4.99% of the proportional variability and had higher loadings for MS (0.66). F8 accounted for 4.18% of the proportional variability and loaded heavily on WW (−0.44) and RLRV (0.79), whereas F9 accounted for 3.80% of the proportional variability and only loaded on RA (0.83). Because the remaining subsequent factors with eigenvalues < 1 explained a small amount of variance, they were not considered for further analysis. The range of communality of variables for the 27 body conformation traits was between 0.39 and 0.72.

### 3.5. Heritability of Factor Scores

Variance components for nine different factor scores using the different relationship matrices are shown in Table 5. Heritability estimates for the pedigree relationship matrix had a mean value of 0.18 with a standard error of 0.08, whereas for the combined genomic-pedigree matrix, the mean value of heritability was 0.20 with a standard error of 0.08 for all considered factor scores. In particular, the lowest heritability estimates were for F2 (feet and legs factor score and muscularity factor score) and F4 based on both methods. However, for both matrices, the highest values of heritability observed were for F1, a factor score accounting for the body frame and rump individual body conformation traits. Factor 3 and Factor 5 (i.e., the mammary system factor score) exhibited medium heritability values based on both matrices. In general, there was no significant difference in the heritability estimates of factor scores obtained using the pedigree relationship matrix or the combined genomic-pedigree matrix.

### 3.6. Correlations between EBV of Composite Traits and EBV of Factor Scores

Results of the Spearman and Pearson correlation analyses (only absolute values ≥  0.20 reported) between EBV of composite traits and EBV of factor scores are reported in Table 6 and Table 7. The results of the Spearman and Pearson correlation analyses were very similar. Correlation coefficients exhibited patterns very similar to the loading coefficients of the individual body conformation traits for F1, F2, and F4. EBVs obtained for F1 were highly positively correlated with the EBVs of the body frame, muscularity, and rump traits. In addition, Spearman and Pearson correlations between EBVs of F2 and EBVs of muscularity and feet and legs-related traits were positive, consistent with results previously reported for the loading coefficients between individual traits and the second latent factor. This pattern was also observed for F4; Spearman and Pearson correlations between EBVs of F4 and EBVs of mammary system-related traits were positive. However, similar results were not observed for F3, F5, F6, F7, F8, and F9.

## 4. Discussion

In this study, the heritability of 6 composite traits and 27 individual body conformations ranged from 0.03 to 0.65. The 24 common latent factors explained 96.13% of the total variation in 27 individual body conformation traits. Heritability estimates for the factor scores ranged from 0.008 to 0.43. The Spearman and Pearson correlation results revealed that the correlation coefficients between the EBVs of the factor scores and the EBVs of the composite traits exhibited a very similar pattern to that of the loading coefficients of the individual body conformation traits for the F1, F2, and F4.

### 4.1. Phenotype

Linear scoring of dual-purpose Simmental cattle has not been conducted in Northwest China. We quantified the body conformation traits that could be reliably measured to develop a linear scoring criterion. However, some individual body conformation traits were difficult to measure, such as rib and bone, rear leg rear view, and fore udder attachment. We scored ten hard-to-measure individual body conformation traits on a 9-point linear scale. The means of all scored traits ranged from 4 to 6. Similar findings were reported by Strapáková et al. [28] and Zavadilová et al. [29]. In addition, similar findings have been reported for Chinese Holstein cattle [19], US Brown Swiss dairy cattle [9], and Rendena and Aosta Red Pied dual-purpose breeds [18].

The mean values for body frame (85.14) and feet and legs scores (86.72) were slightly higher than those for Slovenian Simmental dairy cows (81.35 and 81.06, respectively). In contrast, scores for muscularity (80.72) and the mammary system (78.77) were similar for the two species [28]. For body measurement traits, the mean values of stature (140.65 cm) and body depth (78.41 cm) were slightly lower than those of Slovakia Simmental dairy cows (144.31 cm and 82.92 cm, respectively) [28] and slightly higher than those of Czech Fleckvieh cows (137.40 cm and 77.40 cm, respectively) [29]. In addition, the rump length (51.83 cm) of dual-purpose Simmental cattle in Northwest China was lower than that of Slovakia Simmental dairy cows (53.31 cm) [29] and Czech Fleckvieh cows (52.80 cm) [28]. In general, the body size of dual-purpose Simmental cattle in northwest China needs to be improved.

### 4.2. Heritability

In this study, except for muscularity traits, the composite traits were consistent with those of dual-purpose Rendena cattle [14], dual-purpose autochthonous Valdostana cattle [30], German and French dairy cattle [31], and Czech Fleckvieh cattle [32]. Previous studies [32,33] reported muscularity to be a medium to high heritability trait; this differs from the conclusion based on the results of our study and may be due to different definitions of muscularity. The heritability of individual body frame traits was high (0.11 to 0.65), followed by the rump traits (0.15 to 0.34) and mammary system traits (0.03 to 0.34), and the heritability was low for feet and legs traits (0.07 to 0.16) and muscularity traits (0.04 to 0.09). As reported by Kern et al. [34], Gibson et al. [9], and Spehar et al. [35], RUW and MS are moderately heritable traits (0.12–0.17) in Brazilian Holstein cattle, American Brown Swiss cattle, and Slovenian Brown Swiss cattle, while the results of this study indicated low heritability of these traits (0.04–0.06). Roveglia et al. [36] reported a heritability of 0.07 for RUW in their study of Italian Jersey cattle, which was similar to the results of our study (0.07). The differences in magnitude observed across these studies may be due to the scales used for measurement and scoring, the number of animals, breeds, statistical models, data editing procedures, and consistency among evaluators [37].

In addition, we investigated the heritability for composite traits and individual body conformation traits using the H matrix in dual-purpose Simmental cattle. Comparing the estimates of heritability and their standard errors for each trait using A and H, there was no significant difference for any conformation trait, except for ST. Based on the standard errors, there were no significant differences in any traits. It is possible that the construction of H was primarily determined by the information found in A since there were too few individuals with genotypes. Therefore, they had little influence on the genetic parameter estimates. In addition, the small number of individuals with phenotypic and pedigree records, and the specific environmental effects on farms have influenced the results of the genetic parameter estimation in this study. Some researchers have shown that genomic information can improve the accuracy of genetic parameter estimation for breeding target traits. For example, Veerkamp et al. [38] and Wei et al. [39] demonstrated that rescaling H according to the eigenvalues of A slightly changed the genetic variances. However, there are several reasons why estimated genetic variances differ between the models using pedigree and genomic relationships. First, A and H use different scales for the diagonal elements, especially when considering the Mendelian sampling component in H. A second reason is related to the genetic structure of cattle populations. The third reason is the accuracy of pedigree records. Naserkheil et al. [40] and Song et al. [41] demonstrated that single-step GBLUP provides a more accurate prediction than traditional BLUP for all the studied traits. There are so few individuals with available genotypes in the current study that combining data from genotyped and nongenotyped animals are not worthwhile. In future studies, additional individuals with available genotypes will be included in the genomic analysis of conformation traits in dual-purpose Simmental cattle.

### 4.3. Factor Analysis

In multivariate statistical analysis, factor analysis is one of the classical tools [18,19,42,43]. Several studies have shown that a small number of factors can be used to accurately describe the cow’s conformation without reducing accuracy [18,19,44,45]. An essential aspect of the present study is the algebraic sign and magnitude of the loading coefficients and the percentage of the total variance explained by each factor. A trait with a high loading coefficient contributes more to the factor than one with a low loading coefficient [44]. Once the loading coefficients are determined, with a varimax rotation, it is possible to posit a biological interpretation of the factors [46]. Kaiser introduced the varimax rotation criterion; it maximizes the sum of variances between variables and factors squared [47]. Generally, factor analysis can be understood as a data-reduction technique that removes duplicate information from a collection of correlated variables [43]. The most frequently used factor analysis procedure in the literature has been the matrix transformation step, followed by the extraction of all factors using the principal-factor method with eigenvalues ≥ 1.0 and then rotation of these factors by varimax [18,19,45,48]. Finally, the factors can be explained by identifying the traits with the largest values. Since the procedure is available in many computer statistical packages (e.g., SAS and SPSS), it is relatively easy to use.

Phenotypic factor analysis in this study extracted 24 principal components, which accounted for 96.13% of the total variance among the 27 body conformation traits. Chu and Shi [43] found that eigenvalues > 1 explained 49.1% of the total variance in type traits of Holstein cows in the Beijing area. A similar value was found for the first six latent factors in a study of Aosta Red Pied cattle [18]. The approximate percentage of the total variance explained was determined in a factor analysis of the Rendena breed conducted by Mantovani et al. [48]. The results of the analysis indicated that high values of F1, representing body frame, rump, and muscularity traits, were associated with greater height and buttocks size. High correlations were also noted in previous studies [49,50]. As reported by Manafizar et al. [51], ST, CW, and BD exhibited a strong genetic correlation with residual feed intake, and the work of Dadati et al. [52] indicated that the rump score had the highest genetic correlation with easier calving. Therefore, selection based on F1 may improve the milk production, meat production and reproductive performance of dual-purpose cattle. F2 is characterized by high and positive loading coefficients for heel depth and foot angle and is usually associated with lameness. Thus, the selection of dual-purpose cattle based on high scores for feet and legs, steeper foot angle, straighter legs, and fine bone structure might improve locomotion and lower the risk of claw disorder [53,54]. F3, F4, F5, and F6 were udder trait-related factors, indicating the size and quality of the mammary system, respectively. Mazza et al. [18] reported that F3 and F4 reflected the mammary system in dual-purpose autochthonous breeds. High values of F3 were associated with teat placement, high values of F4 were associated with thick and long teats, high values of F5 were related to large udders, and high values of F6 were associated with shallow, strong, and balanced udders. Researchers have extensively studied the genetic correlation between conformation traits and SCS [9,55,56,57]. The strongest correlations were obtained for FUA, FTP, and UD. F3, F4, F5, and F6 included traits that are usually associated with SCS, and a selection index based on higher udders with tighter attachments and closer teats would be favorable for reducing SCS [58]. Dube et al. [59] found that narrow teat placement and low, shallow udders were strongly correlated with low SCC in the South African Holstein population. Evaluating latent factors instead of original traits is an interesting approach. However, in reality, there is almost no routine application of conducting a genetic evaluation on the factors in any country.

### 4.4. Correlations between EBV of Composite Traits and EBV of Factor Scores

Consistent with the findings of Mantovani et al. [48], the Spearman correlation (rs) analyses between composite trait EBVs and factor score EBVs showed very similar patterns to the loading coefficients of individual traits in latent factors. For instance, the EBVs obtained for F1 exhibited a high correlation with the EBVs of body frame, muscularity, and rump traits (0.55 < rs < 0.67 and 0.59 < r < 0.69). Additionally, correlation between EBVs of F2 and EBVs of feet and legs also showed positive correlations (rs = 0.20 and r = 0.33), and EBVs for mammary system and udder conformation factors (i.e., F4) also showed positive correlations (rs = 0.30 and r = 0.39). The Spearman and Pearson correlation results for F7 and the composite trait EBVs were similar to those of F1, exhibiting high correlations with body frame, muscularity, and rump. Because of the generally high Spearman and Pearson correlations between the EBVs of factor scores and the respective EBVs of body conformation traits associated with those factors, factor scores could be used to guide animal breeding. However, it is crucial to select factors prudently since the random error could attenuate any further analysis based on the newly extracted variable in the factor score [26]. Heritability estimates of the nine factor scores showed that in both matrices, the most heritable factor was related to body frame, muscularity, and rump traits (F1), whereas the least heritable factor was related to feet and legs traits (F2).

The amount of phenotypic and genotypic data collected was small due to the late start of the linear assessment of body conformation and genomic evaluation of dual-purpose Simmental cattle in Northwest China. In the future, we will establish a protocol for type classification in dual-purpose Simmental cattle and conduct annual body conformation linear identification and incorporate it into the breeding programme.

## 5. Conclusions

In this study, the body conformation traits showed a range of heritability from low to high, with stature yielding the highest estimates. The number of animals with recorded body conformation traits is rather low, especially the number of genotyped animals, which led to little difference in the precision of estimating heritability using the pedigree relationship matrix and the combined genomic-pedigree matrix. The factor scores exhibited low to medium heritability, and the generally high Spearman and Pearson correlations between EBVs of Factor 1, Factor 2, and Factor 4 and the corresponding EBVs for composite traits suggested their utility in selection programmes. These analyses suggest that a few factors can describe a variety of body conformation traits without reducing the accuracy of genetic assessments.

## Figures and Tables

**Table 1:** Description of body conformation traits in dual-purpose Simmental Cattle.

Traits	Number	Minimum	Maximum	Average	SD	CV (%)
Composite trait						
Final score (points)	1016	74.6	87.1	82.2	2.1	2.5
Body frame (points)	1016	68.5	95.0	85.1	4.6	5.4
Muscularity (points)	1016	70.5	91.5	80.7	3.1	3.9
Rump (points)	1016	58.0	95.0	80.4	6.2	7.7
feet and legs (points)	1016	73.0	94.3	86.7	3.6	4.2
Mammary system (points)	1016	64.2	88.1	78.8	4.2	5.4
Individual body conformation trait						
Body frame						
Stature (cm)	1016	126	154	140.7	4.5	3.2
Body depth (cm)	1016	61	90	78.4	6.0	7.6
Chest width (cm)	1016	18	39	27.6	4.3	15.6
Muscularity						
Withers width (cm)	1016	10	28	18.4	2.9	15.6
Hind leg half circumference (cm)	1016	33	52	42.5	3.0	6.9
Rear leg height (cm)	1016	62	88	76.6	4.3	5.6
Rib and bone (points)	1016	1	9	6.8	1.2	18.0
Rump						
Rump length (cm)	1016	43	59	51.8	3.1	5.9
Rump width (cm)	1016	17	29	22.7	1.9	8.4
Rump angle (cm)	1016	−5	17	6.3	3.3	52.7
Feet and legs						
Heel depth (cm)	1016	1	8	4.1	0.9	21.7
Foot angle (points)	1016	2	9	5.4	1.1	20.0
Rear legs side view (points)	1016	2	9	5.2	1.2	22.4
Bone quality (points)	1016	4	8	6.1	0.6	9.8
Rear legs rear view (points)	1016	2	9	5.2	1.2	22.4
Mammary system						
Rear Udder height (cm)	1016	14	38	29.0	4.2	14.4
Rear Udder width (cm)	1016	7	24	13.0	2.9	22.4
Median suspensory (cm)	1016	0	8	3.5	1.5	42.9
Udder depth (cm)	1016	−19	22	6.7	5.2	77.2
Fore udder length (cm)	1016	10	29	17.7	3.4	19.2
Front teat length (cm)	1016	2	10	4.8	1.3	27.2
Front teat diameter (cm)	1016	1	4	2.5	0.5	21.3
Fore udder attachment (points)	1016	1	8	4.2	1.3	32.0
Rear udder length (points)	1016	1	9	5.0	1.5	29.7
Udder balance (points)	1016	1	9	4.7	0.9	19.0
Fore teat placement (points)	1016	1	8	4.0	1.1	26.4
Rear teat placement (points)	1016	1	9	5.2	1.0	19.5

**Table 2:** Variance components and heritability of body conformation traits in dual-purpose Simmental Cattle obtained using the pedigree relationship matrix (A) and combined genomic-pedigree relationship matrix (H).

	A Matrix				H Matrix			
Traits ^1^	$\sigma_{a}^{2}$	$\sigma_{e}^{2}$	$\sigma_{p}^{2}$	$h^{2}\;\pm \;SE$	$\sigma_{a}^{2}$	$\sigma_{e}^{2}$	$\sigma_{p}^{2}$	$h^{2}\;\pm \;SE$
Composite trait								
Final score	0.72	3.33	4.05	0.18 ± 0.08	0.55	3.50	4.05	0.14 ± 0.07
Body frame	8.47	11.27	19.74	0.43 ± 0.13	9.36	10.48	19.84	0.47 ± 0.12
Muscularity	0.62	8.86	9.48	0.07 ± 0.05	0.99	8.53	9.52	0.10 ± 0.06
Rump	7.57	23.94	31.51	0.24 ± 0.10	6.65	24.74	31.39	0.21 ± 0.09
Feet and leg	1.29	11.37	12.66	0.10 ± 0.06	1.27	11.41	12.68	0.10 ± 0.06
Mammary system	2.74	10.63	13.37	0.20 ± 0.09	3.11	10.33	13.44	0.23 ± 0.09
Average	3.57	11.57	15.14	0.20 ± 0.09	3.66	11.50	15.15	0.21 ± 0.08
Individual body conformation								
Body frame								
ST	11.38	8.93	20.31	0.56 ± 0.12	13.48	7.17	20.66	0.65 ± 0.11
BD	5.27	25.33	30.60	0.17 ± 0.08	6.00	24.70	30.70	0.20 ± 0.09
CW	2.29	15.72	18.01	0.13 ± 0.08	2.04	15.96	18.00	0.11 ± 0.07
Average	6.31	16.66	22.97	0.29 ± 0.09	7.17	15.94	23.12	0.32 ± 0.09
Muscularity								
WW	0.77	7.51	8.28	0.09 ± 0.07	0.53	7.73	8.26	0.06 ± 0.06
HLHC	0.53	7.55	8.08	0.07 ± 0.05	0.64	7.45	8.09	0.08 ± 0.05
RLH	0.86	14.95	15.81	0.05 ± 0.05	0.94	14.88	15.82	0.06 ± 0.05
RAB	0.07	1.29	1.36	0.05 ± 0.05	0.52	1.31	1.83	0.04 ± 0.04
Average	0.56	7.83	8.38	0.07 ± 0.06	0.66	7.84	8.50	0.06 ± 0.05
Rump								
RL	2.24	5.57	7.81	0.29 ± 0.11	2.67	5.23	7.90	0.34 ± 0.12
RW	0.68	2.40	3.08	0.22 ± 0.09	0.62	2.46	3.08	0.20 ± 0.08
RA	1.39	7.85	9.24	0.15 ± 0.07	1.94	7.35	9.29	0.21 ± 0.08
Average	1.44	5.27	6.71	0.22 ± 0.09	1.74	5.01	6.76	0.25 ± 0.09
Feet and legs								
HD	0.03	0.59	0.62	0.05 ± 0.05	0.05	0.58	0.63	0.07 ± 0.05
FA	0.11	0.83	0.94	0.11 ± 0.06	0.15	0.78	0.93	0.16 ± 0.07
RLSV	0.11	1.18	1.29	0.09 ± 0.06	0.09	1.20	1.29	0.07 ± 0.05
BQ	0.02	0.32	0.34	0.07 ± 0.05	0.04	0.30	0.34	0.12 ± 0.06
RLRV	0.19	1.34	1.53	0.12 ± 0.07	0.20	1.33	1.53	0.13 ± 0.07
Average	0.09	0.85	0.94	0.09 ± 0.06	0.11	0.84	0.94	0.11 ± 0.06
Mammary system								
RUH	3.08	14.72	17.80	0.17 ± 0.07	3.00	14.82	17.82	0.17 ± 0.07
RUW	0.57	7.06	7.63	0.07 ± 0.05	0.83	6.81	7.64	0.11 ± 0.06
MS	0.11	1.73	1.84	0.06 ± 0.05	0.18	1.67	1.85	0.10 ± 0.06
UD	4.01	14.15	18.16	0.22 ± 0.09	4.71	13.55	18.26	0.26 ± 0.09
FUL	1.02	7.91	8.93	0.11 ± 0.06	1.08	7.87	8.95	0.12 ± 0.07
FTL	0.21	1.57	1.78	0.12 ± 0.06	0.20	1.58	1.78	0.11 ± 0.06
FTD	0.05	0.23	0.28	0.18 ± 0.07	0.05	0.23	0.28	0.18 ± 0.07
FUA	0.31	1.34	1.65	0.19 ± 0.08	0.44	1.23	1.67	0.27 ± 0.09
RUL	0.14	1.75	1.89	0.07 ± 0.06	0.05	1.83	1.88	0.03 ± 0.05
UB	0.22	0.61	0.83	0.26 ± 0.10	0.28	0.54	0.82	0.34 ± 0.09
FTP	0.19	0.78	0.97	0.20 ± 0.08	0.20	0.78	0.98	0.20 ± 0.08
RTP	0.16	0.76	0.92	0.17 ± 0.08	0.20	0.72	0.92	0.22 ± 0.09
Average	0.84	4.38	5.22	0.15 ± 0.07	0.94	4.30	5.24	0.18 ± 0.07

^1^ ST: stature; BD: body depth; CW: chest width; WW: withers width; HLHC: hind leg half circumference; RLH: Rear leg height; RAB: Rib and bone; RL: rump length; RW: rump width; RA: rump angle; HD: heel depth; FA: foot angle; RLSV: rear legs side view; BQ: Bone quality; RLRV: rear legs rear view; RUH: rear udder height; RUW: rear udder width; MS: median suspensory; UD: udder depth; FUL: fore udder length; FTL: fore teat length; FTD: fore teat diameter; FUA: fore udder attachment; RUL: rear udder length; UB: udder balance; FTP: fore teat placement; RTP: rear teat placement; $\sigma_{a}^{2}$: additive genetic variance; $\sigma_{e}^{2}$: residual variance; $\sigma_{p}^{2}$: phenotype variance; $h^{2}$: heritability; SE: standard error.

**Table 3:** Eigenvalues and proportion of total and cumulative variance explained by factor analysis of the body conformation traits in dual-purpose Simmental cattle.

Factor	Eigenvalue	Proportional Variance (%)	Cumulative Variance (%)
F1	3.65	13.51	13.51
F2	2.20	8.13	21.65
F3	1.98	7.32	28.96
F4	1.62	6.01	34.97
F5	1.40	5.18	40.15
F6	1.33	4.93	45.08
F7	1.21	4.49	49.57
F8	1.13	4.18	53.75
F9	1.03	3.80	57.55
F10	0.96	3.56	61.12
F11	0.93	3.46	64.58
F12	0.89	3.30	67.88
F13	0.86	3.18	71.05
F14	0.81	3.01	74.06
F15	0.74	2.74	76.81
F16	0.73	2.73	79.54
F17	0.71	2.65	82.19
F18	0.67	2.47	84.66
F19	0.62	2.29	86.94
F20	0.59	2.17	89.11
F21	0.56	2.09	91.20
F22	0.49	1.81	93.00
F23	0.43	1.60	94.60
F24	0.41	1.52	96.13

**Table 4:** Latent factors, loading of individual body conformation traits (loading coefficients ≥$\;\left|0.40\right|$), and communality after varimax rotation of the 27 body conformation traits in dual-purpose Simmental cattle.

	Varimax Latent Factors									
Trait ^1^	F1	F2	F3	F4	F5	F6	F7	F8	F9	Communality
ST	0.65									0.68
BD	0.46						−0.51			0.68
CW							−0.45			0.39
WW								−0.44		0.54
HLHC	0.60									0.43
RLH		0.46								0.54
RAB					−0.42					0.44
RL	0.72									0.62
RW	0.57									0.49
RA									0.83	0.72
HD		0.76								0.64
FA		0.77								0.67
RLSV										0.44
BQ										0.46
RLRV								0.79		0.68
RAH						−0.53				0.52
RUW					0.58					0.60
MS							0.66			0.51
UD						0.50				0.63
FUL					0.70					0.60
FTL				0.80						0.68
FTD				0.81						0.72
FUA						0.53				0.51
RUL					0.50					0.55
UB						0.65				0.48
FTP			0.79							0.63
RTP			0.73							0.68
Variance explained (%)	2.32	1.98	1.91	1.85	1.69	1.68	1.52	1.33	1.26	

^1^ ST: stature; BD: body depth; CW: chest width; WW: withers width; HLHC: hind leg half circumference; RLH: Rear leg height; RAB: Rib and bone; RL: rump length; RW: rump width; RA: rump angle; HD: heel depth; FA: foot angle; RLSV: rear legs side view; BQ: Bone quality; RLRV: rear legs rear view; RUH: rear udder height; RUW: rear udder width; MS: median suspensory; UD: udder depth; FUL: fore udder length; FTL: fore teat length; FTD: fore teat diameter; FUA: fore udder attachment; RUL: rear udder length; UB: udder balance; FTP: fore teat placement; RTP: rear teat placement.

**Table 5:** Estimated variance components, heritability and standard errors for 9 factor scores obtained using the pedigree relationship matrix (A) and combined genomic-pedigree relationship matrix (H).

	A Matrix				H Matrix			
Factor Score	$\sigma_{a}^{2}$	$\sigma_{e}^{2}$	$\sigma_{p}^{2}$	$h^{2}\;\pm \;SE$	$\sigma_{a}^{2}$	$\sigma_{e}^{2}$	$\sigma_{p}^{2}$	$h^{2}\;\pm \;SE$
F1	0.39	0.52	0.91	0.43 ± 0.13	0.39	0.52	0.91	0.43 ± 0.12
F2	0.04	0.64	0.68	0.06 ± 0.05	0.07	0.62	0.69	0.10 ± 0.06
F3	0.16	0.61	0.77	0.21 ± 0.08	0.16	0.62	0.78	0.21 ± 0.09
F4	0.008	0.92	0.93	0.008 ± 0.04	0.04	0.90	0.94	0.04 ± 0.05
F5	0.20	0.71	0.91	0.22 ± 0.10	0.24	0.68	0.92	0.26 ± 0.11
F6	0.09	0.70	0.79	0.11 ± 0.07	0.11	0.68	0.79	0.14 ± 0.08
F7	0.06	0.60	0.66	0.09 ± 0.05	0.07	0.59	0.66	0.10 ± 0.06
F8	0.08	0.58	0.66	0.12 ± 0.06	0.10	0.57	0.67	0.15 ± 0.07
F9	0.31	0.58	0.89	0.35 ± 0.12	0.34	0.55	0.89	0.38 ± 0.12

**Table 6:** Spearman correlation coefficients (only values ≥$\;\left|0.20\right|$) between EBVs estimated for body conformation traits and EBVs obtained from 9 factor scores (from F1 to F9) in dual-purpose Simmental cattle.

Composite Traits	F1	F2	F3	F4	F5	F6	F7	F8	F9
Body frame	0.67			−0.31			0.28		
Muscularity	0.55	0.24		0.23	0.20	−0.24	0.45		
Rump	0.58		0.24		0.23		0.44		
feet and legs		0.20							
Mammary system				0.30				0.31	0.44

**Table 7:** Pearson correlation coefficients (only values ≥ $\left|0.20\right|$) between EBVs estimated for body conformation traits and EBVs obtained from 9 factor scores (from F1 to F9) in dual-purpose Simmental cattle.

Composite Traits	F1	F2	F3	F4	F5	F6	F7	F8	F9
Body frame	0.69			−0.34			0.36		
Muscularity	0.59	0.31		0.23	0.22	−0.25	0.58		
Rump	0.64		0.33		0.28		0.51		
feet and legs		0.33							
Mammary system				0.39				0.32	0.50

## Data Availability

The data that support the findings of this study are available from the corresponding author, L.X., upon reasonable request.
